# The Devil Is in the Details

**DOI:** 10.1007/s10508-022-02513-2

**Published:** 2023-01-06

**Authors:** Julia Bahner, Don Kulick, Andrea García-Santesmases Fernández

**Affiliations:** 1 School of Social Work, Lund University, Box 23, 221 00 Lund, Sweden; 2 Department of Cultural Anthropology and Ethnology, Uppsala University, Uppsala, Sweden; 3 Department of Social Work, National University of Distance Education (UNED), Madrid, Spain

Benoit et al.’s (2022) Target Article neatly summarizes available literature about access to sexual support for people with a range of disabilities. The scoping review is both useful and timely, considering the sudden bloom of non-medical research around sexuality and disability. Recent monographs such as *Loneliness and Its Opposite* (Kulick & Rydström, 2015), *Already Doing It* (Gill, 2015), *The Intimate Lives of Disabled People* (Liddiard, 2018), *Sexual Citizenship and Disability* (Bahner, 2020), and *Narrowed Lives* (Vehmas & Meitola, 2021) show that sexuality and disability studies finally have taken a decisive step away from a purely medical or rehabilitative register to become an interdisciplinary, multi-perspectival, international research field. The anthologies *Routledge Handbook of Disability and Sexuality* (Shuttleworth & Mona, 2021) and *Diverse Voices of Disabled Sexualities in the Global South* (Chappell & de Beer, 2019) cement this move. They explore a wide range of impairments, different gendered, sexual, and racial identities, and the range, possibilities, and limitations of support and health services that people with disabilities encounter. These books, together with many of the articles discussed in the scoping review, exemplify the heterogeneity and vitality of studies that have emerged in the wake of the seminal work *The Sexual Politics of Disability: Untold Desires* (Shakespeare et al., 1996). The Target Article by Benoit et al. is a further welcome contribution to this increasingly robust field.

Sexual support is a dimension of sexuality that inevitably emerges as soon as discussion focusses on people with disabilities. As Benoit et al. (2022) rightly point out, sexual support is a deeply contentious issue. It is fraught with moral, ethical, legal, and practical dilemmas which vary greatly across cultural and sociopolitical contexts (Sakairi, 2020; Wotton, 2021). Reasons why different types of sexual support services emerge are related to: (1) whether disability rights movements and other agents such as social workers, politicians, and the parents of disabled people have advocated for sexual rights, or not; (2) what types of mainstream sexual services are available (and legal) in society; and (3) culturally specific views of disability and the place of disabled people in society (Bahner, 2020; Kulick & Rydström, 2015).

Benoit et al.’s (2022) classification of the research they review into “sex-negative” vs. “sex-positive” perspectives is not unreasonable, but this is well-tread ground and one wonders if there are any questions at all about sexuality that can’t be treated in such a framework. In order to move the field of sexuality and disability forward, we would have appreciated a more nuanced contextual categorization of the work under review, namely one that highlighted the structural, organizational, and practical constraints and possibilities that exist for the “population of interest” under discussion. It would have been helpful if Benoit et al. had framed their discussion in terms of where exactly sex work is legal and where it is not. For example, they could have addressed whether particular places have any explicit policies or guidelines for disability services regarding sexual support—or, indeed, if there even are any comprehensive disability support services in the first place.

In addition, Benoit et al. (2022) get certain facts wrong. For instance, they mention Spain as one of the countries that “have embedded facilitated sexual assistance into their public disability policies.” This is not the case. Even though there are multiple sexual assistance organizations in the country, the support they provide exists in a legally ambiguous vacuum (García-Santesmases Fernández & Branco de Castro Ferreira, 2016). Similarly, in the Netherlands, although sex work is legalized, the so-called sex care organizations are neither legally acknowledged nor embedded in national policy (Bahner, 2020).

While the tables in the Target Article contain information about the focus of the included articles (for example, “ethical debate,” “literature review,” “empirical research,” and so on), this information is not always made explicit in the discussion, which makes it difficult to determine whether the arguments in the cited articles involved collaboration with sexual support workers, or with people with disabilities, disability rights organizations, parents, etc. It seems clear that the articles which support what Benoit et al. (2022) call “sex-negative perspectives” often are primarily ethical debates, in some cases written by polemicists who are antagonistic toward sex work, who have little contact or experience with people with disabilities, or who are careful to write only about abuse in ways that imply that all sexual activity involving adults with disabilities, by definition, is abusive. It seems to us that this kind of distinction—where writers largely unconcerned with people with disabilities dilate about them and writers connected in some way to disability worlds engage with disability affirmatively—is unsurprising and even obvious: After all, “Nothing About Us Without Us” is a venerable disability rights slogan.

A further problem with the dichotomous labeling adopted in the Target Article is that a label like “sex-positive” obscures important complexities that are worth articulating and exploring. Articles discussing “sex surrogates,” for example, are classified as “sex-positive,” but this is contentious. Many people with disabilities regard the idea that sex with people with disabilities ought to require special training or certification as patronizing and medicalizing (e.g., Kulick & Rydström, 2015, pp. 184–186; García-Santesmases Fernández & Branco de Castro Ferreira, 2016, pp. 16–20; García-Santesmases, 2022, pp. 171–174; Shakespeare et al., 1996, pp. 132–134). Perhaps a more trenchant classification for the work reviewed by Benoit et al. (2022) would have been between research focused on developing disabled people’s lives and research that uses disabled people primarily as proxies to address other issues, such as sex work. Their conclusions seem to make precisely this point.

It is important to note that what sexual assistance means exactly is frequently not clear, neither in practice nor in theory. The organizations, services, and people that provide sexual support services are few, and the rationale for the services—in addition to what exactly is offered and enacted—is all so different as to be unique (Bahner, 2020; García-Santesmases Fernández & Branco de Castro Ferreira, 2016; Kulick & Rydström, 2015). While Benoit et al.’s (2022) Target Article acknowledges that such differences exist (for example, in Table 1), the differences themselves are not detailed or explored further. Our view is that the variety and range of support services, in itself, is profoundly consequential and deserves detailed attention. Even disabled activists who regard themselves feminist and sex-positive have diverse and sometimes opposing views on sexual assistance. Some frame sexual assistance as a medical/rehabilitative remedy, based on ideas about the normalization of the disabled body. Others align with crip approaches, where a function of sex is to challenge heteronormative ideals of a “normal” body and acceptable sexuality (García-Santesmases, 2022; García-Santesmases Fernández et al., 2017). There is a great deal more at stake here than “cultural scripts” that are either “sex-negative” or “sex-positive.”

A final complexity that we wish had been foregrounded more clearly when thinking of sexual support is the role that understandings about specific disability types can play in how a disabled person’s sexuality is regarded by policy makers, support managers and staff, family members, and others who have an impact on such individuals’ sexual lives. The relative intensity of an impairment, whether it is congenital or acquired, physical, sensory or cognitive, and so on, necessarily will influence what type of sexual support is appropriate (Bahner, 2021). Although the Target Article includes type of impairment as a category, and it notes that the situation for people with intellectual disabilities usually occurs in “sex-negative perspectives,” other kinds of differences are not discussed in any detail. Benoit et al. point out that dimensions of diversity, such as gender and ethnic background, are often missing in the articles they include, but readers of their scoping review would have benefitted from their reflections on whether diversity among people with disabilities is discussed in the included articles, and what such discussion might mean for their arguments and for future research.

We applaud Benoit et al. (2022) for having gathered together and summarized literature with which we all will want to consult and engage. While we have critiqued their classification of the literature in terms of “cultural scripts” that use “sex-negative” and “sex-positive” perspectives on sexuality and disability, we want to allow that Benoit et al. perhaps have done us all a service. Those labels, while they may be useful as classificatory schemes or political slogans, are clearly dead-ends as analytic tools. To move forward—to understand sexuality in the lives of people with a variety of disabilities and the people who interact with, care for, and love them—we need finer understandings of context. A finer understanding of context will also help to refine our thinking about interventions that might improve those lives and make them even better and more vital. We need to move away from generalities like “sex-positivity” or “sex-negativity” and pay close and respectful attention to the granular detail of everyday lives in situated contexts. One of the many services this scoping review may perform is to close the chapter on generalizations and open a new chapter, where we all get to work and document specificities.
